# Prenatal conditions do not affect brain physiology and learning in a lizard

**DOI:** 10.1242/jeb.250716

**Published:** 2025-08-12

**Authors:** Pablo Recio, Dalton C. Leibold, Ondi L. Crino, Christopher R. Friesen, Daniel W. A. Noble

**Affiliations:** ^1^ Division of Ecology and Evolution, Research School of Biology, The Australian National University, Canberra, ACT 2601, Australia; ^2^ Flinders University, College of Science and Engineering, Bedford Park, SA 5042, Australia; ^3^ University of Wollongong, Wollongong, NSW 2500, Australia; ^4^ Environmental Futures University of Wollongong, Wollongong, NSW 2500, Australia

**Keywords:** Cognition, Corticosterone, Incubation temperature, Learning, Mitochondria, Reptiles

## Abstract

Early environmental factors such as heat or stress hormones can impair learning through brain metabolic function, which is crucial for neural development and synaptic plasticity. However, whether early environments always result in cognitive impairment through changes in neural physiology is not well established outside of a few model systems. Here, we investigated the effects of prenatal temperature and corticosterone (CORT) on brain mitochondrial activity and spatial learning in the delicate skink (*Lampropholis delicata*). We treated eggs with either CORT or a control vehicle and incubated at cold (23±3°C) or hot (28±3°C) temperatures. Juveniles were tested in a spatial learning task over 40 days after which mitochondrial function in the medial cortex was assessed. Despite among-individual variation in learning ability, mitochondrial physiology and spatial learning in *L. delicata* remained robust to prenatal temperature and CORT exposure. No significant relationship was found between mitochondrial function and cognitive performance, contrary to predictions. Increased metabolic capacity correlated with higher production of reactive oxygen species but did not affect oxidative damage, possibly as a result of protective mechanisms. These findings highlight the physiological and cognitive resilience of *L. delicata* to early-life challenges. Future research should explore whether this robustness extends to other brain regions, cognitive domains and life stages.

## Teaser

**Summary:** Unlike mammals, the delicate skink (*Lampropholis delicata*) shows no effects of prenatal stress or temperature on brain mitochondrial function or cognition-related metabolism.

## INTRODUCTION

Learning – the acquisition and consolidation of new information – enables animals to create new associations between events, which can be essential for coping with environmental change (Buchanan et al., 2013; Dukas, 2004; Leal and Powell, 2012). However, the capacity to form new associations varies among individuals, potentially affecting their responses to environmental challenges (Ward-Fear et al., 2016; Welklin et al., 2024). Learning faster may imply better exploitation of resources or more efficient responses to novel threats (Ward-Fear et al., 2016). Nevertheless, learning fast can also carry potential costs, such as reduced memory accuracy or energetic trade-offs (Dukas, 2004). As such, variation in learning abilities likely reflects different evolutionary compromises, with significant implications for individual fitness (Ward-Fear et al., 2016; Welklin et al., 2024). Unravelling the mechanisms that drive these differences is crucial for understanding cognitive evolution.

Factors such as age, sex or early-life conditions can have important effects on learning abilities (Amiel and Shine, 2012; Amiel et al., 2014; Carazo et al., 2014; Lemaire et al., 2000; Noble et al., 2014; Szuran et al., 1994; Zhu et al., 2004). Adverse developmental environments are particularly influential, as the brain is highly sensitive to environmental inputs during early life (Zhu et al., 2004). For example, early experiences can alter neurotransmitter production (Amani et al., 2021), gene expression (Zhou et al., 2020) or brain structure (Amiel et al., 2017), with lasting effects on cognition. Among the physiological mechanisms underlying learning, mitochondrial activity is considered a key factor (Du et al., 2009; Picard and McEwen, 2014; Picard et al., 2018; Siegel and Albers, 1994). The neural processes involved in learning impose substantial energetic demands (Alexandrov and Pletnikov, 2022; Mann et al., 2021; McNay et al., 2000), making efficient mitochondrial respiration essential (Du et al., 2009; Picard and McEwen, 2014; Picard et al., 2018). Additionally, learning depends on a dense network of functional neurons (Amiel et al., 2017; Lefebvre, 2011), which can be compromised by excessive oxidative stress – a byproduct of mitochondrial metabolism (Du et al., 2009; Finkel and Holbrook, 2000; Gong et al., 2011; Hoffmann and Spengler, 2018; Zhu et al., 2004). Studies on mammals have shown the pervasive effects of mitochondrial physiology on cognitive abilities (Cao et al., 2014; Hara et al., 2014; Zhu et al., 2004). However, the extent to which these effects are generalisable to other taxa – particularly reptiles – remains largely unexplored (see Matsubara et al., 2017; Szabo et al., 2021).

As mitochondria are maternally inherited, maternal condition plays a fundamental role in shaping offspring mitochondrial activity (Picard and McEwen, 2014). Maternal stress can also influence how mitochondria operate in offspring (Zhu et al., 2004). Stressful situations experienced by mothers can elevate glucocorticoids (GCs) (Sapolsky et al., 2000), which can affect developing embryos (Uller et al., 2009), altering mitochondrial physiology through transgenerational effects (Picard and McEwen, 2014). For instance, maternal stress has been shown to contribute significantly to oxidative stress in the brain of rats (*Rattus norvegicus*) with impacts on spatial learning abilities (Cao et al., 2014; Haussmann et al., 2012; Zhu et al., 2004).

Temperature is also a significant source of maternal stress in ectotherms. Thermal environments outside the optimal range can elevate GCs in mothers, which can be passed to the offspring (see Crino et al., 2023). Temperature can also directly influence offspring development, particularly during early life (Crino et al., 2024; Noble et al., 2018). The prenatal thermal environment is crucial in shaping mitochondrial function, affecting energy metabolism and oxidative stress (Crino et al., 2024; Stier et al., 2022). Thus, the combined effects of prenatal GCs and temperature may profoundly influence mitochondrial function, with important consequences for brain development and cognition. However, the extent to which prenatal GCs and temperature interact to shape cognitive abilities via mitochondrial physiology remains largely unknown outside of a few model species (Cao et al., 2014; Haussmann et al., 2012; Zhu et al., 2004).

Here, we examined how prenatal temperature and corticosterone (CORT) – the primary GC in reptiles – affect brain mitochondrial physiology and spatial learning in the delicate skink (*Lampropholis delicata*). We hypothesised that prenatal CORT and temperature would influence learning abilities by impacting brain mitochondrial activity. Specifically, we predicted that prenatal CORT would decrease energy production while increasing oxidative damage (Costantini et al., 2011; Gong et al., 2011; but see Crino et al., 2024), especially if CORT can alter cellular components increasing reactive oxygen species (ROS) production without enhancing ATP synthesis or making these cellular components more vulnerable to oxidative damage. Similarly, we predicted that high temperatures would decrease mitochondrial efficiency (Crino et al., 2024; Závorka et al., 2021), but would reduce oxidative stress (Treidel et al., 2016). We further predicted that the combined effects of CORT and temperature would lead to complex interactions, with both factors negatively impacting mitochondrial efficiency but having opposite effects on oxidative stress. These effects would lead to differences in learning abilities, which could be affected by the balance between energy production and oxidative stress (Alexandrov and Pletnikov, 2022; Du et al., 2009; Picard et al., 2018). By examining these interactions, we aimed to clarify how prenatal environmental factors shape learning abilities through mitochondrial function, clarifying the mechanisms that mediate the role of early-life conditions on cognitive development.

## MATERIALS AND METHODS

### Ethics

Both the breeding animals and the experimental lizards were provided with humane laboratory housing, with thermoregulatory opportunities, light (UV and heat) and moderate humidity levels (see Supplementary Materials and Methods, ‘Animal husbandry’, for details). Euthanasia was performed by intraperitoneal injection of 10 mg kg^−1^ of a 10 mg ml^−1^ alfaxan solution (a potent anaesthetic) followed by decapitation. We monitored the animals to ensure no irritation from the agent as indicated by distressed animals. Before disposing of the lizard, we confirmed the absence of righting response and the pinching reflex in one of the front limbs. All the protocols complied with Australian law and were approved by the Australian National University Animal Experimentation Ethics Committee (A2022_33).

### Experimental animals

**Fig. 1.**
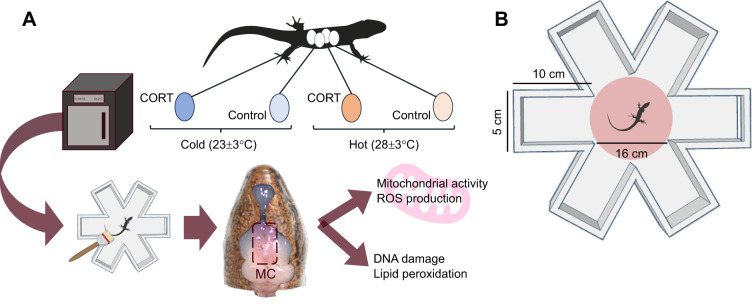
**Schematic diagram of our experimental design.** (A) The different stages of our experiment and the main manipulations. CORT, corticosterone; ROS, reactive oxygen species; MC, medial cortex. (B) The spatial learning maze (dimensions indicated), showing the initial position of the lizard (*Lampropholis delicata*) in the centre.

*Lampropholis delicata* (De Vis 1888) came from a breeding colony established in the laboratory in 2019 from wild populations in Sydney, Australia. The colony consisted of 270 adults in groups of two males and four females. Eggs were collected from these groups between November 2023 and January 2024. The collected eggs belonged to either the first or second generation of our breeding colony. After collecting the eggs, we treated them with CORT or vehicle control and incubated them under two different temperature regimes (see below). Clutch and egg identity were assigned immediately after egg collection, and the eggs were incubated in individual cups until hatching. Hatchlings were also kept in individual enclosures until the end of the experiment (see Fig. 1A). For details on husbandry and breeding conditions, see Supplementary Materials and Methods, ‘Animal husbandry’.

### CORT and temperature manipulations

We tested the combined effects of prenatal CORT and temperature by elevating CORT concentrations in eggs and then incubating them under one of two temperature regimes (cold 23±3°C or hot 28±3°C). We used a partial split clutch design where eggs from a given clutch were distributed equally across the four treatments when clutch sizes were larger than four and randomly across treatments when less than four. CORT-treated eggs were topically supplied with 5 µl of crystalline corticosterone (Sigma, cat. no. C2505) dissolved in 100% ethanol at a final concentration of 10 pg CORT ml^−1^ (CORT treatment), while control eggs received an equal volume of 100% ethanol. This CORT dose increased mean yolk CORT levels 2 standard deviations higher than those of control eggs in previous studies (Crino et al., 2024). Eggs were then incubated in one of the two previously mentioned temperature regimes. Mean incubation temperatures represent the lower and upper limits of the natural range of nest temperatures in *L. delicata* (mean nesting temperature 27.4°C; Cheetham et al., 2011). Incubation temperatures fluctuated daily by ±3°C with all eggs within a treatment group experiencing identical thermal cycles mimicking natural conditions. Although the absolute temperature ranges of the two regimes overlapped slightly (e.g. 25–26°C), the thermal cycles were synchronised such that the same temperatures were never experienced at the same time between treatments. Nonetheless, recent meta-analyses suggest little difference between daily temperature fluctuation treatments and constant ones (Raynal et al., 2022; Stocker et al., 2024). The higher temperature treatment (28°C) was above the thermal optima estimated for *L. delicata* (*T*_opt_=25°C; Pettersen et al., 2023) and reached more stressful temperatures daily as fluctuations occurred around the mean treatment temperature (i.e. 31°C).

### Spatial learning

The spatial learning task involved training lizards to navigate a 6-arm maze (see Fig. 1B) to reach an exit connected to a transport box that allowed us to return the lizards to their enclosure without further contact. In each trial, lizards were placed by hand in the centre of the maze and left to acclimatise for 2 min. During acclimatisation, the central area was surrounded by a yellow device mounted on a pulley system. At the start of each trial, this device was lifted to startle the lizard. If the lizard did not immediately choose an arm, it was gently prodded with a brush at the end of the tail. A lizard was considered to have made a choice once it inserted its head into one of the arms. If the chosen arm was incorrect, we encouraged movement by gently touching the lizard with a brush, but without guiding it toward any specific arm or back to the centre of the maze. Each incorrect choice was recorded as an error, regardless of whether the same arm had been visited previously. If the lizard did not choose the correct arm after 20 errors – at which point it typically ceased responding – it was gently guided to the correct arm.

We assessed lizards' spatial learning using external cues while avoiding intra-maze cues. External cues included a flag constructed from sticks and fabric placed in a consistent position outside the maze. In addition, the position of the overhead light and the experimenter remained fixed throughout the experiment and may have also served as orientation references. Subtle intra-maze cues were avoided by replacing the maze every three trials with one of four identical mazes. Each replacement preserved the correct arm's orientation and the maze's position within the room, ensuring consistency for each individual. Additionally, the maze was cleaned with 70% ethanol between trials to eliminate chemical cues. The correct arm was randomly assigned to one of the six arms for each lizard to control for potential side bias. We employed four maze orientations and counterbalanced the number of lizards assigned to each orientation across treatments.

The task was repeated once daily for 40 consecutive days. By the end of the experiment, lizards were between 44 and 76 days (mean 62.6 days) post-hatching. All lizards were allowed to thermoregulate freely in their home enclosure before each trial, which was performed during the hours of peak activity (10:00–14:00 h). For each trial, we recorded the number of errors made, which served as the response variable in the learning analyses. We would expect the number of errors to decrease significantly over time if lizards were learning.

### Brain mitochondrial function

Immediately after completing the tests, we quantified mitochondrial physiology in the brains of the lizards (see Fig. 1A). We euthanised lizards via intraperitoneal injection of 10 mg kg^−1^ of a 10 mg ml^−1^ alfaxan solution (a potent anaesthetic), followed by decapitation. Before decapitation, we evaluated lizard righting responses and the pinching reflexes in one of the front limbs.

We extracted the medial cortex in the telencephalon as this brain region is considered homologous to the mammalian hippocampus, where spatial cognition is encoded (Naumann et al., 2015; Rodríguez et al., 2002). The tissue was transferred to 1× PBS solution and then homogenised mechanically using a 100 µm mesh filter (pluriStrainer). The resultant homogenate was divided into two aliquots: one was used fresh for measuring mitochondrial density, membrane potential – a metric of mitochondrial metabolic capacity (Martínez-Reyes et al., 2016) – and superoxide (ROS) production; and the other was cryopreserved for later measurement of DNA damage and lipid peroxidation.

Fresh homogenate suspensions were stained with 5 µmol l^−1^ MitoTracker Deep Red FM, 2.5 µmol l^−1^ MitoTracker Orange CMTMRos and 50 µmol l^−1^ MitoSOX Red. We used these fluorescent probes to assess mitochondrial density, membrane potential and ROS, respectively. We also added 5 µl of 10 µg ml^−1^ Hoechst 33342 Nuclear Viability Dye to each sample to differentiate viable cells from debris. These samples were analysed in a flow cytometer (details below) within 2 h of brain extraction.

The aliquots reserved for oxidative damage assays were stained with 10 µg ml^−1^ Hoechst 33342 Nuclear Viability Dye and 100 µmol l^−1^ BODIPY 665/676 Lipid Peroxidation Sensor before cryopreservation. These dyes were used to measure cell viability and lipid peroxidation, respectively. The samples were then fixed in 1% neutral-buffered formalin, washed, and preserved in a 1× Tris-EDTA solution with 10% DMSO at −20°C. On the day of the oxidative damage assays, the samples were thawed and the DMSO was removed. Then, cell membranes were permeabilised in 200 µl of 1× PBS containing 20 µmol l^−1^ digitonin. Following permeabilization, we stained the samples with 20 µl of 70 µmol l^−1^ 8-OHdG polyclonal antibody (BS-1278R, ThermoFisher) – a marker of DNA damage – and allowed them to incubate overnight at 4°C. The following day, we counterstained the cells with 20 µl of 100 µg ml^−1^ H+L goat anti-rabbit conjugate antibody with Alexa-Fluor 488 (A27012, ThermoFisher) and analysed the samples using the flow cytometer (see below). Oxidative damage assays were performed within 6 months of the initial preparation of fresh samples.

Flow cytometry was performed on a 5-laser flow cytometer (Becton Dickson LSRFortessa X-20) using the default wavelength filters and a high-throughput plate reader. Detectors and voltage settings for each assay were determined during pilot trials and remained consistent throughout the experiment. Data were processed using FlowJo (v.10.1) software. We obtained the mean fluorescence intensity for mitochondrial density, metabolic capacity, ROS, DNA damage and lipid peroxidation. For further details on the homogenisation, staining and flow cytometry assays, see Supplementary Materials and Methods, Flow cytometry. Sample sizes for DNA damage and lipid peroxidation were smaller than for the other variables because of a plater reader malfunction; however, data were missing randomly and we dealt with missing data in our analyses (see below). We validated that our homogenates contained neurons in a pilot study that used dyes specifically targeting neuronal nuclei (see Supplementary Materials and Methods, Brain validation). This pilot study also ensured that our gating strategy identified these neurons using flow cytometry.

### Statistical analyses

We performed all analyses using the *brm* package (Bürkner, 2017), which fits Bayesian multilevel models with *Stan* (https://mc-stan.org/rstan/) with R version 4.4.0 (http://www.R-project.org/). We ran a series of univariate and multivariate models to test the effects of early environment on each variable separately plus quantify the relationships between physiology and learning. All models consisted of four MCMC chains of 8000 iterations, with a warmup interval of 2000 iterations.

Univariate models were used to test the effects of early environment on each variable recorded: mitochondrial density, metabolic capacity, ROS, DNA damage, lipid peroxidation and the number of errors as a measure of learning. In all the models, we included hormone treatment (CORT versus control), incubation temperature (cold versus hot), and their interaction. Sex and age were included in preliminary models and excluded from the final models when they were not significant. For all univariate models, clutch identity was included as a random factor. Mitochondria-related variables were log-transformed when necessary, and all were mean-centred and standardised by dividing them by 2× standard deviation (Gelman, 2008). These variables followed a normal distribution.

Learning was modelled as a function of trial, CORT, temperature and their three-way interaction. In this model, we included the trial within each level of lizard identity as a random slope. The error structure was modelled using a negative binomial distribution with a logit link function [negbinomial(link=‘log’)]. Otherwise, the procedure was as in the other models.

We used the posterior distributions of parameters from these models to test for differences between treatments. Learning slopes were obtained using the ‘trial’ estimates and their interaction with hormone and temperature treatments. Slope estimates less than 0 provide evidence of learning. *P*_MCMC_ was used to test the hypothesis that posterior distributions of slopes and slope contrasts differed from zero. We considered an effect statistically significant when *P*_MCMC_<0.05.

We fitted a multivariate structural equation model (SEM) using *brms* to explore direct and indirect links between mitochondrial function and learning. The model was structured based on theoretical expectations shown in Fig. 4. Any missing data were imputed during model fitting using data augmentation (Noble and Nakagawa, 2021), but this was largely restricted to DNA damage and lipid peroxidation. To obtain a measure of learning performance, we extracted the posterior distribution of individual learning slopes (i.e. changes in error over time) by including a random trial slope for each lizard. Learning slopes and the rest of variables were standardised as before (see above). We used Gaussian error distributions for all the variables in this model. Factors found to be non-significant in the univariate models were excluded. Clutch identity remained a random factor in our SEM model. Direct, indirect and total effects were derived from posterior estimates (Kline, 2005) (see Table S4).

### Declaration of AI use

We declare ChatGPT-4 was used for questions related to code checking when there were unknown errors, but we did not rely on AI for the creation of any of the content in the manuscript, which was written fully by the authors. The last time that Chat GPT-4 was used was February 2025.

For an alternative representation of the data and model checks, see Table S10 and Figs S1–S8.

## RESULTS

We started with 80 lizards – 20 per treatment – from 33 clutches. However, because of natural mortality, only 79 lizards were included in the learning analyses, and 78 in the mitochondrial analyses. We provide final sample sizes in the figures.

Univariate models suggested some variation between clutches for all the response variables as summarised in Table S3. We also found some variation across individuals in learning rates (s.d. trial slope=0.204; 95% confidence interval, CI=[0.035, 0.343]).

**Fig. 2.**
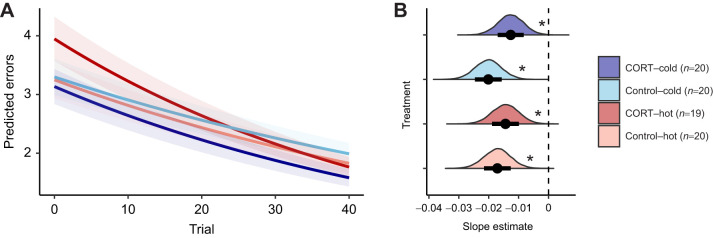
**Model estimates for learning analyses.** (A) The predicted number of errors over trials. The lines represent the mean predicted number of errors for each trial, and the shaded areas indicate the s.e.m.; both were obtained using the slope and intercept estimates from the posterior distributions. (B) The distribution of the estimates of slopes for each treatment. The *x*-axis represents the slope estimate and the *y*-axis shows the density of the estimates. Circles and bars represent the mean and standard error of the estimated slopes, respectively. Dashed lines indicate a value of 0. The different colours indicate the different treatments. Asterisks in B indicate significant differences from 0 (*P_MCMC_<0.05).

We found that the lizards improved their choices over time, making fewer mistakes as the trials progressed (see Fig. 2; Table S1). However, the learning rate was not influenced by CORT, temperature or their interaction (see contrasts in Table S2).

**Fig. 3.**
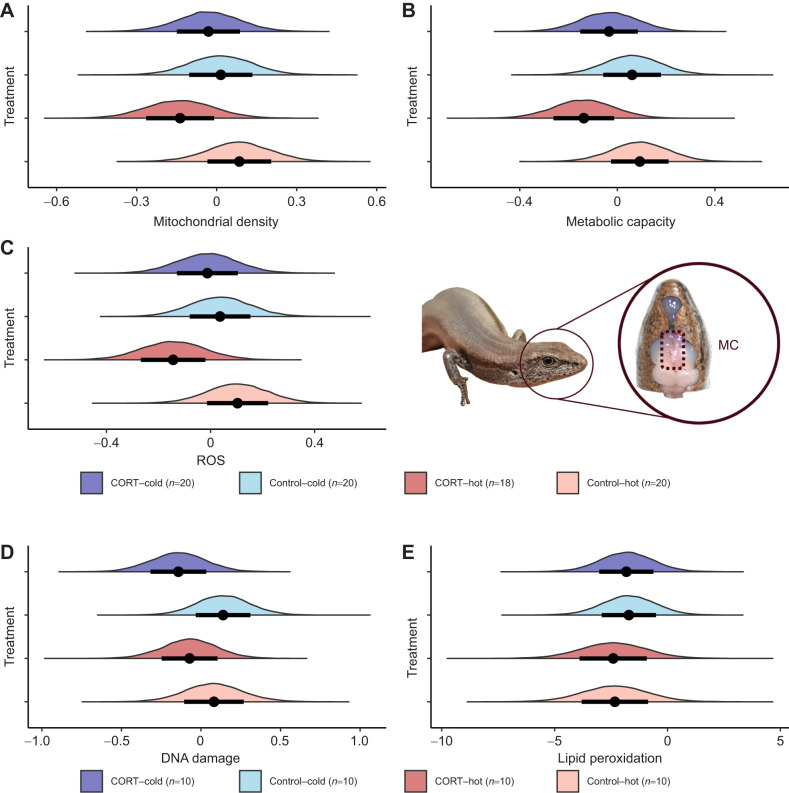
**Model estimates of brain mitochondrial function.** (A) Mitochondrial density, (B) metabolic capacity, (C) ROS, (D) DNA damage and (E) lipid peroxidation in the medial cortex (MC) as a function of the different prenatal conditions. Note that for D and E, these analyses do not account for missing data resulting from a flow cytometer malfunction that impacted one plate, so sample sizes are lower for univariate analyses. The *x*-axis represents the estimated values and the *y*-axis shows the density of the estimates. Circles and bars represent the mean and standard error of the estimated values, respectively. The different colours indicate the different treatments.

Mitochondrial density, metabolic capacity, ROS, DNA damage and lipid peroxidation were also not affected by prenatal conditions (see Fig. 3; Table S2). We did not find any effects of sex and age on mitochondrial physiology (see Tables S5–S9).

**Fig. 4.**
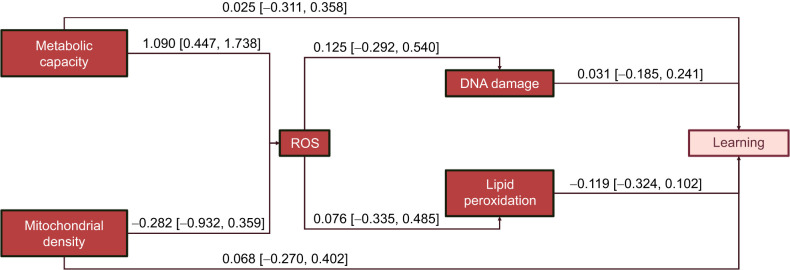
**Structural equation model testing hypothesised direct and indirect effects of physiology on learning.** Arrows indicate the directionality of the estimate, and the values show the mean predicted coefficient and its 95% confidence interval.

Our SEM showed that mitochondrial physiology was unrelated to learning abilities (see Fig. 4; Table S4). While we found that ROS production increased with metabolic capacity (β=1.090, 95% CI=[0.316, 1.862], *P*_MCMC_<0.05), ROS was not related to oxidative damage (see Fig. 4; Table S4).

## DISCUSSION

Lizards were able to learn a complex spatial task with marked improvements over time in their ability to choose the correct location. However, learning was neither influenced by prenatal conditions nor linked to mitochondrial function in the medial cortex, counter to what we predicted. Despite ROS increasing with greater metabolic capacity as expected, this did not result in a corresponding increase in oxidative damage in the medial cortex in *L. delicata*. We discuss our results in relation to existing work exploring the underlying mechanisms of learning and how early environments impact it below.

### Spatial learning is robust to prenatal conditions

Lizards exhibited clear evidence of learning, but learning rates were not influenced by prenatal CORT or temperature as we predicted, contrasting with findings in other taxa where incubation temperature and prenatal GCs impact learning and brain development (Amiel and Shine, 2012; Amiel et al., 2017; Dayananda and Webb, 2017; Lemaire et al., 2000; Zhu et al., 2004). Our results seem to suggest that spatial learning abilities in *L. delicata* is buffered from adverse early environmental conditions, consistent with our previous findings showing that behavioural flexibility in this species is also unaffected by prenatal CORT or temperature (Recio et al., 2025). *Lampropholis delicata*’s resilience may stem from the relevance of spatial cognition in lizards. For example, spatial learning in velvet geckos (*Amalosia lesueurii*) enhances survival in the wild (Dayananda and Webb, 2017).

Alternatively, the experimental treatments may not have been sufficiently challenging to elicit detectable effects, and the apparent resilience observed in *L. delicata* may reflect the relatively mild nature of the prenatal conditions. However, our CORT treatment elevated CORT levels in eggs above 2 standard deviations of the natural mean concentration (Crino et al., 2024), while our thermal regimes were both beyond the *T*_opt_ in this species (Pettersen et al., 2023) and yet within the range of nesting temperatures in the wild (Cheetham et al., 2011). Therefore, we consider that our manipulations represent biologically relevant, ecologically realistic stressors. Nonetheless, future studies could explore whether more extreme conditions, such as prolonged exposure to elevated CORT or incubation temperatures beyond the natural nesting limits would affect brain function and cognition in *L. delicata* or other lizard species.

### Brain metabolic physiology is not affected by prenatal conditions

Contrary to our predictions, prenatal temperature and CORT did not significantly influence mitochondrial physiology in the medial cortex of *L. delicata*. Studies in other taxa have shown that incubation at high temperatures decreases energy production and oxidative stress (Treidel et al., 2016; Závorka et al., 2021), while elevated GCs or maternal stress is related to lower mitochondrial efficiency and higher oxidative damage (Costantini et al., 2011; Gong et al., 2011; but see Crino et al., 2024). In *L. delicata*, high incubation temperatures are known to decrease mitochondrial efficiency in the liver (Crino et al., 2024). However, our study shows that mitochondrial physiology in *L. delicata*’s brain is robust to both incubation temperature and prenatal CORT. Differences between liver and brain could reflect tissue-dependent responses to early-life conditions, highlighting the importance of studying these effects across multiple tissues (Casagrande et al., 2023).

### Variation in brain metabolic physiology is not related to learning abilities

Mitochondria play a critical role in cognitive function by synthesising energy (Du et al., 2009; Picard and McEwen, 2014; Picard et al., 2018; Siegel and Albers, 1994), and by affecting the rate of cell senescence and death caused by oxidative damage (Alexandrov and Pletnikov, 2022; Mann et al., 2021; McNay et al., 2000). Our findings contrast with past studies and suggest that energy limitations do not impact spatial learning in *L. delicata*. The lack of association between mitochondrial density, mitochondrial capacity and learning abilities may result from lizards being able to maintain energy production above the threshold required to prevent cognitive dysfunction. The generally low energetic demands of ectothermic organisms compared with those of endotherms may make this possible and explain why we found no effect when they seem common in mammals (Cao et al., 2014; Hara et al., 2014). Alternatively, individuals with higher respiration and ROS may be upregulating antioxidant production (Rice et al., 2002).

Interestingly, despite our finding of a significant positive relationship between metabolic capacity and ROS in the medial cortex, the heightened ROS production did not lead to greater oxidative damage. The strong relationship between ROS and mitochondrial capacity aligns with the role of mitochondrial membrane potential in driving ATP synthesis and electron transport, processes that inherently generate ROS as byproducts (Rice et al., 2002). Normally, moderate ROS levels serve essential signalling functions and are controlled by antioxidants (Rice et al., 2002; Terman and Brunk, 2006). However, excessive ROS production can exceed antioxidant action, leading to oxidative stress and cellular dysfunction (Rice et al., 2002; Terman and Brunk, 2006). The lack of oxidative damage from increased ROS strongly suggests that antioxidants could deal with these levels of ROS. As such, DNA damage and lipid peroxidation in the medial cortex were not linked to spatial learning in *L. delicata* possibly because neurons were effectively buffered from damaging free radicals. Nevertheless, as oxidative damage can accumulate over time (Terman and Brunk, 2006), the relationships between oxidative damage and cognitive dysfunction could become more pronounced in older individuals (Hara et al., 2014). Future research should investigate the long-term consequences of mitochondrial activity on brain health and the potential mechanisms that sustain cognitive resilience in *L. delicata*.

### Conclusions

We found that spatial learning in *L. delicata* was not influenced by prenatal CORT or temperature, nor was mitochondrial physiology in the medial cortex. Additionally, there was no relationship between mitochondrial function and learning abilities in a spatial task. However, we found that ROS production increased with metabolic capacity, suggesting that links between mitochondrial respiration and oxidative stress may become more pronounced under higher energetic demands or with ageing, potentially influencing cognitive function over time.

## Supplementary Material

10.1242/jexbio.250716_sup1 Supplementary information
