# Correction: Prenatal conditions do not affect brain physiology and learning in a lizard

**DOI:** 10.1242/jeb.252707

**Published:** 2026-05-12

**Authors:** Pablo Recio, Dalton C. Leibold, Ondi L. Crino, Christopher R. Friesen, Daniel W. A. Noble

There was an error in *J. Exp. Biol.* (2025) **228**, jeb250716 (doi:10.1242/jeb.250716).

The Author contributions statement is incorrect. The correct version is shown below and both the online full-text and PDF versions of the article have been updated.

Conceptualization: P.R., D.C.L., O.L.C., C.R.F., D.W.A.N.; Data curation: P.R., D.C.L.; Formal analysis: P.R., D.C.L.; Funding acquisition: C.R.F., D.W.A.N.; Investigation: P.R., D.C.L.; Methodology: P.R., D.C.L., O.L.C., C.R.F., D.W.A.N.; Project administration: P.R., D.C.L.; Resources: D.W.A.N.; Supervision: D.W.A.N., C.R.F.; Validation: P.R., D.C.L., D.W.A.N.; Visualization: P.R., D.C.L.; Writing – original draft: P.R., D.C.L.; Writing – review & editing: P.R., D.C.L., O.L.C., C.R.F., D.W.A.N.

The authors apologise for these errors and any inconvenience they may have caused.

