# Supplementary material for: Prenatal conditions do not affect brain physiology and learning in a lizard
Source: J Exp Biol. 2025 Aug 12;228(15):jeb250716. doi: 10.1242/jeb.250716 (PMC12401535; doi:10.1242/jeb.250716)
Supplement: Supplementary information [file jexbio-228-250716-s1.pdf]

Supplementary Materials and Methods

Animal husbandry

Breeding colony: Juveniles of *L. delicata* came from a breeding colony established in the laboratory since 2019. This colony consisted of 270 adults housed in plastic containers (41.5 L x 30.5 W x 21 H cm) with six lizards (two males and four females) per enclosure. Enclosures were provided with shelter, nonstick matting, and several small water dishes. The lizards were fed approx. 40 mid-size crickets (*Acheta domestica*) per enclosure three days a week, and water was given daily. The crickets were dusted with calcium weekly and multivitamin and calcium biweekly. Room temperatures were set to 22-24 ºC, but we also provided the enclosures with a heat chord and a heat lamp following a 12 h light:12 h dark cycle keeping warm side of enclosures is usually at 34 ºC.

Eggs collection and incubation: Between mid-November 2023 to mid-January 2024, we placed a small box (12.5 L x 8.3 W x 5 H cm) with moist vermiculite in one side of the communal enclosures to provide females with a place to lay the eggs. These boxes were checked three days a week. After egg collection, we measured length and width with a digital caliper to the nearest 0.1 mm and weighted the eggs with a digital scale ± 0.001g error. Then eggs were treated with CORT or vehicle (see CORT and temperature manipulation below) and were placed in individual cups (80 mL) with moist vermiculite (12 parts water to 4 parts vermiculite). The cups were covered with cling wrap to retain moisture and left in two incubators at two different temperatures (see CORT and temperature manipulation below) until hatching.

Hatchlings: Incubators were checked three times a week for hatchlings. Lizards were measured and weighed immediately after hatching. snout-vent length (SVL) and tail length (TL) were measured to the nearest millimeter, and weight was recorded using a digital scale with an accuracy of ± 0.001 g. Hatchlings were then placed in individual enclosures (18.7L x 13.2W x 6.3H cm) with nonstick matting and a small water dish. Watering, feeding, and temperature conditions were maintained as for adults (see above).

Journal of Experimental Biology • Supplementary information

Flow cytometry

Homogenates: After the medial cortex was extracted, it was transferred immediately to 1.5mL centrifuge tubes containing 100µL of cold 1X PBS and kept on ice until further processing. The tissue was mechanically homogenized by placing the tissue in the well of a 100µm mesh filter (pluriStrainer) affixed atop a 1.5mL centrifuge tube, then mashed with the rubber end of an insulin syringe stopper. The resulting homogenate was then rinsed through the filter with 1000µL of cold 1X PBS to prepare a homogenate suspension. Following homogenization, we centrifuged each sample at 1000 RCF for 10 minutes to pellet cells, then removed the supernatant (hereafter, this process referred to as 'washing') and resuspended the cells in 500µL 1x PBS. This step was performed to remove cellular debris from homogenates.

From each 500µL suspension of homogenate collected on a given trial day, we first added 100µL of homogenate to a pooled sample of each tissue type to use for single-color controls, and the remaining 400µL of homogenate was split among two 200µL aliquots. One aliquot was used fresh to measure mitochondrial function (mitochondrial density, membrane potential, ROS), one aliquot was cryopreserved for later measurements of oxidative damage (8-OHdG, lipid peroxidation), and the third aliquot was cryopreserved for a different experiment.

Staining fresh samples: From fresh homogenate suspensions, we loaded the wells of a 96-well flat-bottom plate (Nunclon) with 50µL of homogenate in duplicates (2 wells per homogenate). To each replicate well, we added 5µL of a fluorescent probe mix containing equal parts 5µM MitoTracker Deep Red FM, 2.5µM MitoTracker Orange CMTMRos, and 50µM MitoSOX Red. We used these fluorescent probes as indicators of mitochondrial density, mitochondrial membrane potential, and superoxide (ROS) production, respectively. We then added 5µL of 10 µg/mL Hoechst 33342 Nuclear Viability Dye to each sample, which we used to distinguish live,

Journal of Experimental Biology • Supplementary information

viable, intact cells from cellular debris. We then loaded 6 wells with 50µL of homogenate taken from each pooled homogenate suspension (12 wells total), which were to be negative and single- color controls. One well was left unstained as a negative control, one was stained with all the probes to be a positive control, and the remaining four wells were treated with 5µL of one of 5µM MitoTracker Deep Red FM, 2.5µM MitoTracker Orange CMTMRos, 50µM MitoSOX Red, or 10 µg/mL Hoechst 33342 Nuclear Viability Dye. Any remaining pooled homogenate was fixed and frozen as previously described. We incubated the loaded plate at 32 °C for 30 minutes to stain and then diluted the samples with 50µL cold 1x PBS to halt the staining process. Upon the completion of staining, samples were immediately transferred to flow cytometry
facilities for data collection and were sampled within 2 hours. Samples prepared this way
remained viable for flow cytometry for approximately 5 hours post-staining at room temperature (~19° C) before cells began rapidly degrading (DCL, personal observation).

The aliquots destined to the analysis of oxidative damage were stained with 20µL (10µL for controls) of 10 µg/mL Hoechst 33342 Nuclear Viability Dye and 20µL of 100µM BODIPY
665/676 Lipid Peroxidation Sensor and incubated at 32°C for 20 minutes. Following staining, we washed cells to prevent further binding of unbound fluorescent probes, then resuspended the pellet and continue with cryopreservation.

Cryopreservation: Aliquots were fixed first by adding the samples into a 1mL solution of 1% Neutral-Buffered Formalin (as a fixative agent) and incubating them at 32°C for 20 min. Then washed the samples and resuspended the cells in 1mL cold 1X Tris-EDTA (chelates metals that can damage DNA during freezing) and 10% DMSO (a cryoprotectant). Samples were stored at - 20°C until oxidative damage assays.

Journal of Experimental Biology • Supplementary information

Staining cryopreserved samples: Assays of oxidative damage from cryopreserved samples were performed within 6 months of the initial processing and analysis of fresh samples. On the day of oxidative damage assays, we rapidly thawed frozen samples by briefly (1-2 minutes) submerging them in hot water. We washed each thawed sample twice, the first time resuspending the pelleted cells in 1000µL warm 1X Tris-EDTA, and the second time in 200µL warm 1X PBS containing 20µM digitonin. We incubated the samples at 32°C for 20 minutes to permeabilize the cell membrane, after which we washed the homogenate and resuspended the pelleted cells in 200µL 1X PBS. We added 20µL of 70µM 8-OHdG Polyclonal Antibody to each sample, and we left the homogenate overnight (~12 hours) for the antibody to bind to 8-OHdG, a marker of oxidative damage on DNA. The following day we counterstained the cells with 20µL of 100 µg/mL H+G Goat Anti-Rabbit Conjugate Antibody with Alexa-Fluor 488 at 32°C for 20 minutes. After the cells had been tagged with 8-OHdG antibodies and counterstained, we washed the cells once more and resuspended the pellet in 400µL of 1X PBS. Unstained and single-color controls were treated identically to samples, but stained with only up to one of BODIPY 665/676, Hoechst 33342, 8-OHdG antibody, or Alexa-Fluor 488 conjugate. Additionally, one control was stained with both 8-OHdG antibody and the Alexa-Fluor 488 conjugate. We then loaded a 96-well plate with 100µL of each single-color control and 100µL in triplicate of each sample. We performed all flow cytometry assays on samples within 48-hours of thawing the samples.

Flow cytometry: All flow cytometry assays were performed using a flow cytometer with 5- lasers (blue, red, yellow-green, violet, and ultraviolet), 20 detectors, and a high-throughput plate reader (Becton Dickson LSRFortessa X-20) using the default wavelength filters on detectors. Immediately prior to all assays, we performed a quality-control check and laser alignment using

Journal of Experimental Biology • Supplementary information

the CS&T function of BD FACSDiva (v. 8.0.1) and BD CS&T fluorescent beads (Lot No. 30664) diluted at 1 drop to 150µL 1X PBS. During data collection, data for single-color controls was filtered using a liberal threshold of 200 on the FSC (roughly, cell size) detector, while data from samples was filtered using a threshold of 200 on the BUV-496 (Hoechst 33342) detector. These thresholds were chosen to filter small debris or inviable or non-intact cells from our observations. The detectors and voltage settings used in data acquisition for each assay type (mitochondrial function, oxidative damage) were determined during pilot trials prior to assays and were not changed during assays to allow for comparison among different plates and samples throughout the experiment. Voltages were chosen to center the distribution of observations in each channel at 10^3 fluorescent intensity and reduce observations of off-scale ($<10^1$ or $>10^5$) events. For the mitochondrial function assay, we recorded data from the following channels (in brackets: voltage; parameter): FSC (44; forward scatter), SSC (180; side scatter), Alexa-Fluor 488 (544; autofluorescence), BUV-496 (450; Hoechst 33342), APC (647; MitoTracker Deep Red FM), PE (522; MitoTracker Orange CMTMRos), and PerCP-Cy5-5 (592; MitoSOX Red) channels. For the oxidative damage assay, we recorded data from the following channels: FSC (425; forward scatter), SSC (300; side scatter), Alexa-Fluor 488 (275; 8-OHdG Antibody + Alexa-Fluor 488 conjugate), BUV-496 (525; Hoechst 33342), and PE-Cy5 (850; BODIPY 665/676). Fluorescent intensity data was collected via the BD FACSDiva (v. 8.0.1) software, with no compensation applied during data collection, and all on a linear scale (detectable range of 0-252166). We recorded data for both the area and height of the fluorescent signal, but only used the area in downstream analyses, with height being recorded for the sake of quality control. Data was exported from BD FACSDiva as individual *.fcs (“flow cytometry standard”) files for each sample, then imported into FlowJo (v. 10.1) for processing.

Data processing: In FlowJo v. 10.1 we first transformed all fluorescent data to a logarithmic base 10 scale, then applied a basic gating process across all channels by filtering to observations within the detectable range (10^1-10^5) to remove any off-scale events. We then used a backgating process wherein we aimed to identify the approximate FSC (cell size) and SSC (cell complexity) range of viable cells that were positive for all stains. We primarily used the BUV-496 channel (Hoechst 33342) in the backgating process to identify intact, nucleated cells (BUV-496 > 10^3). For the mitochondrial function assay, we aimed to identify populations of viable cells containing mitochondria (APC > 103) and actively respiring (PE and PerCP-Cy5-5 > 10^3). For the oxidative damage assay, we aimed to identify populations of cells exhibiting both DNA damage (Alexa- Fluor 488 > 103) and lipid peroxidation (PerCP > 10^3). When backgating was done, we filtered the data to the FSC by SSC range that captured the ideal population. We used the backgated population for compensation of fluorescent spillover between different fluorescent probes. To account for fluorescent spillover, we used a traditional compensation matrix using the compensation function of FlowJo v.10.1. We identified the "positive" population for each channel as the brightest ~2.5% of the distribution of observations in the respective single-color control for that channel and used unstained controls as a universal negative. We visually inspected the compensation matrix and its effects on population distributions for under- and over-compensation, whereupon we changed the compensation matrix manually until data was properly compensated. We applied the compensation matrix to all samples for downstream processing. Following compensation, we again gated the data following the same process as for backgating but using the compensated parameters for each channel. Following gating, we exported the geometric mean (mean fluorescent intensity; MFI) and robust confidence-values for each channel for each sample. For analysis, we exported summary statistics of only the area of the fluorescent signal. Although we exported robust confidence values for checking repeatability between replicate samples, we used the geometric means for each individual as our main response variables in analyses.

Journal of Experimental Biology • Supplementary information

Brain validation

To ensure that neurons were not unintentionally lost during homogenization, we performed a pilot study where we euthanized four lizards and prepared medial cortex homogenates using the same procedures as before. However, to ensure we could identify neurons, homogenates here were dyed with marker that specifically targeted neuron nuclei (Farrow et al., 2021; Storks et al., 2023). We employed fluorescence microscopy and flow cytometry to check for the presence of neurons in the homogenates.

Euthanasia and homogenization for each of brain region followed the procedures outlined above (see *Flow cytometry* above). Before staining the samples, cells were also fixed and permeabilized as described previously (see *Flow cytometry* above). Following permeabilization, we centrifuged the samples at 1000 RCF for 10 minutes and resuspended the pellet in 100 µL of a 1:100 dilution of NeuN + Alexa488 fluorescent conjugate to dye the neuronal nuclei (Farrow et al., 2021; Storks et al., 2023). The samples were incubated at +4 ºC overnight.

The following day we centrifuged the samples at 1000 RCF for 10 minutes and resuspended the pellet in 100 µL of PBS. Samples were split in two: 50 µL from each was reserved for examination under a Zeiss AxioObserver Z1 microscope, while the remaining 50 µL from each sample was pooled into a single tube for flow cytometry analysis. . Flow cytometry of stained cells allowed us to validate that our gating strategy was correct.

Samples examined under the microscope showed a clear presence of neuronal nuclei in the homogenates (Figure S8). Flow cytometry analysis also confirmed the presence of neuronal nuclei in the homogenates, that were similar to the size of the cells employed in the experiment (mean size cells experiment = 2.765; 95% CI = [2.093, 3.139], n = 80; size neurons = 3.977495).

Journal of Experimental Biology • Supplementary information

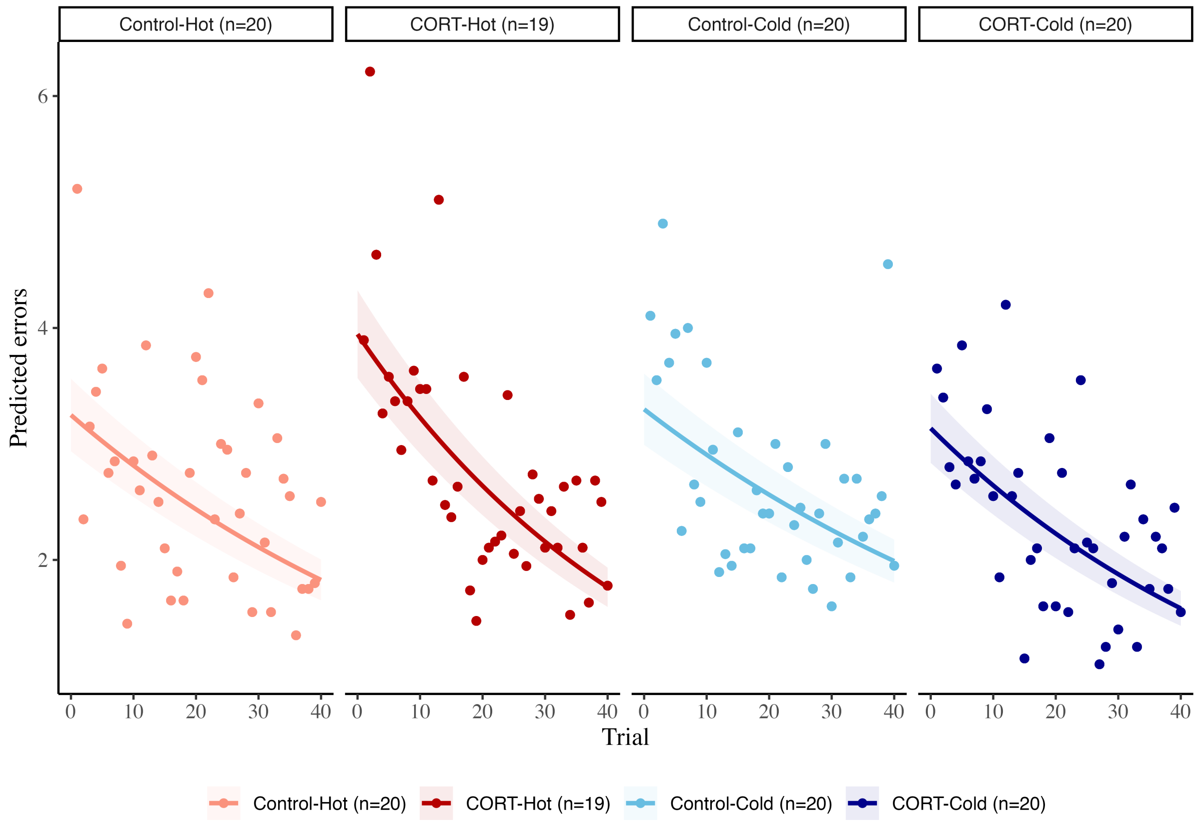

Fig. S1. Raw data of the learning task. Each point represents the mean number of errors per day. The lines represent the modeled data.

Journal of Experimental Biology • Supplementary information

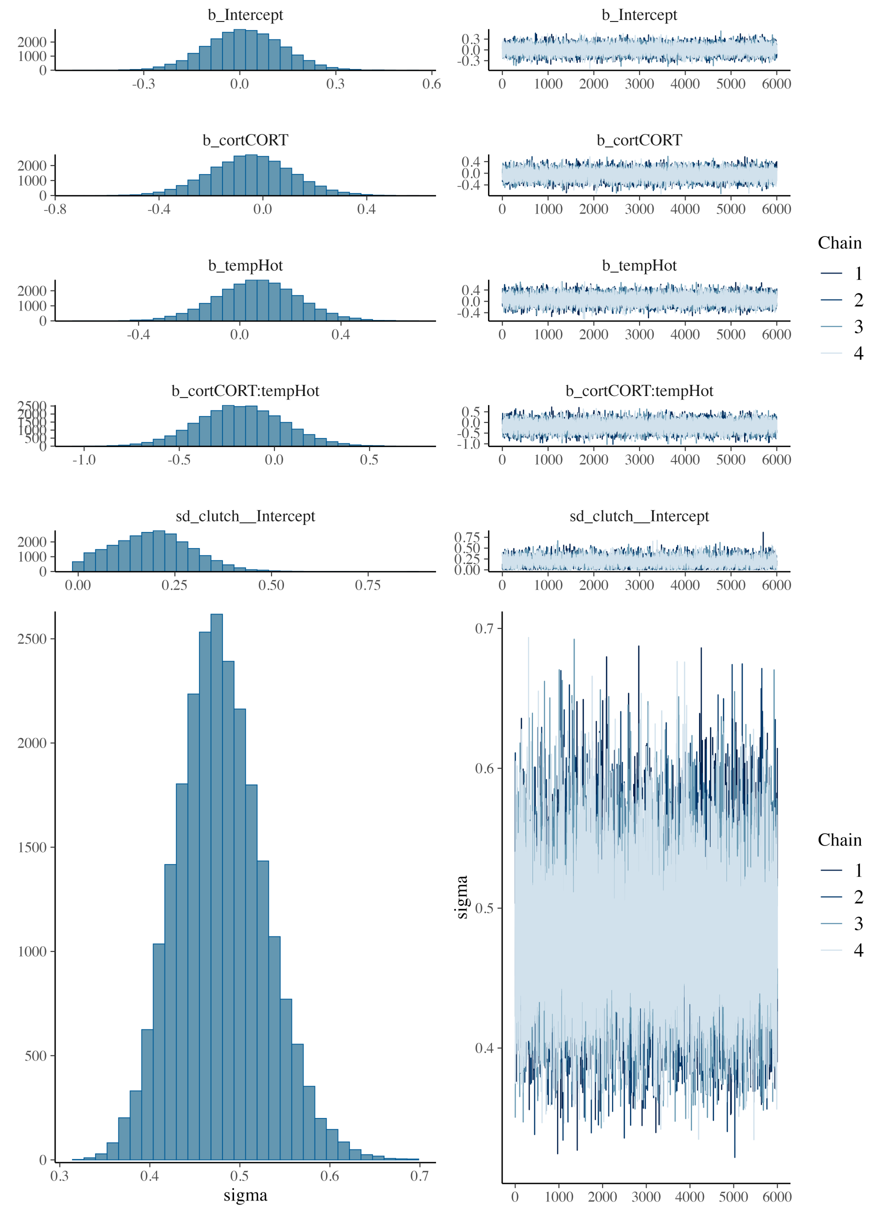

Fig. S2. Posterior predictive checks for the model of Mitochondrial Density. Formula: mit_density ~ cort * temp + (1|clutch)

Journal of Experimental Biology • Supplementary information

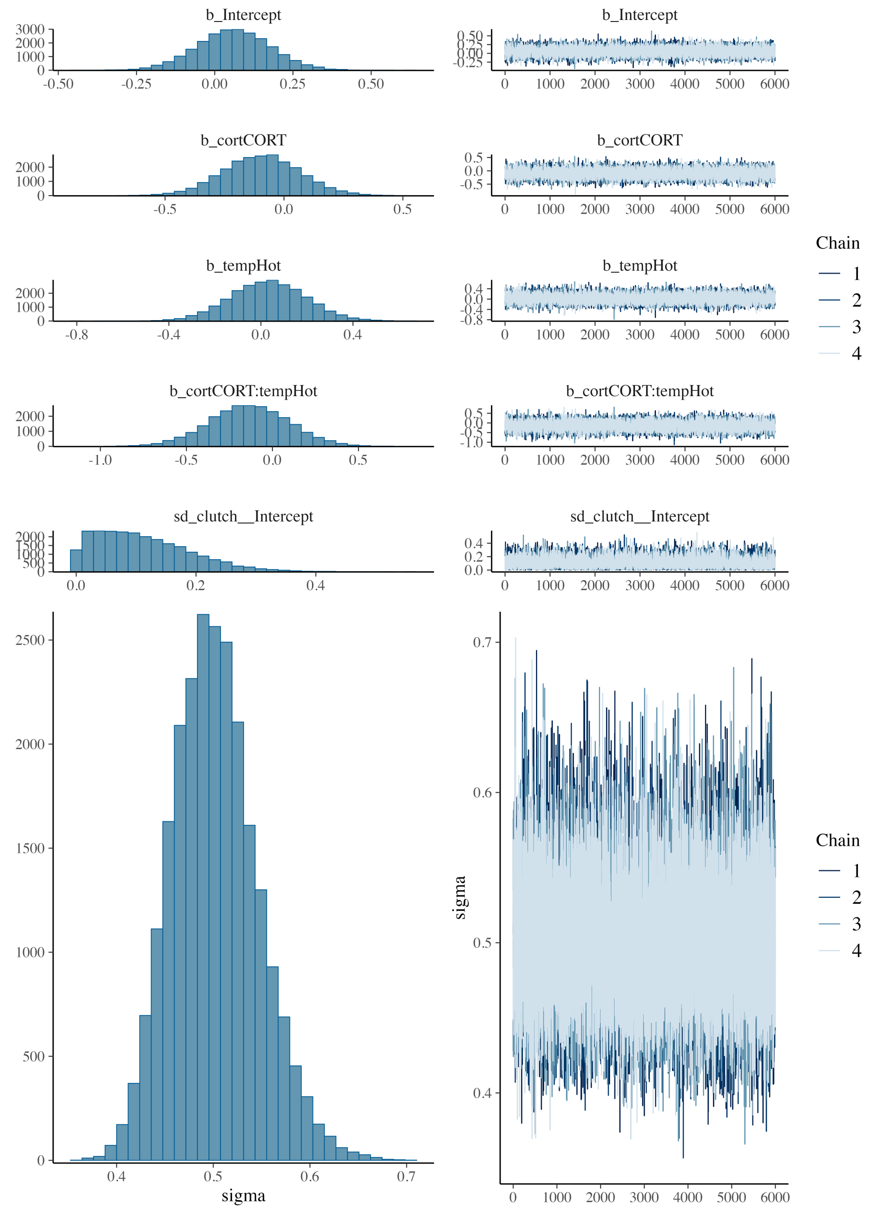

Fig. S3. Posterior predictive checks for the model of metabolic capacity. Formula: mit_potential ~ cort * temp + (1|clutch)

Journal of Experimental Biology • Supplementary information

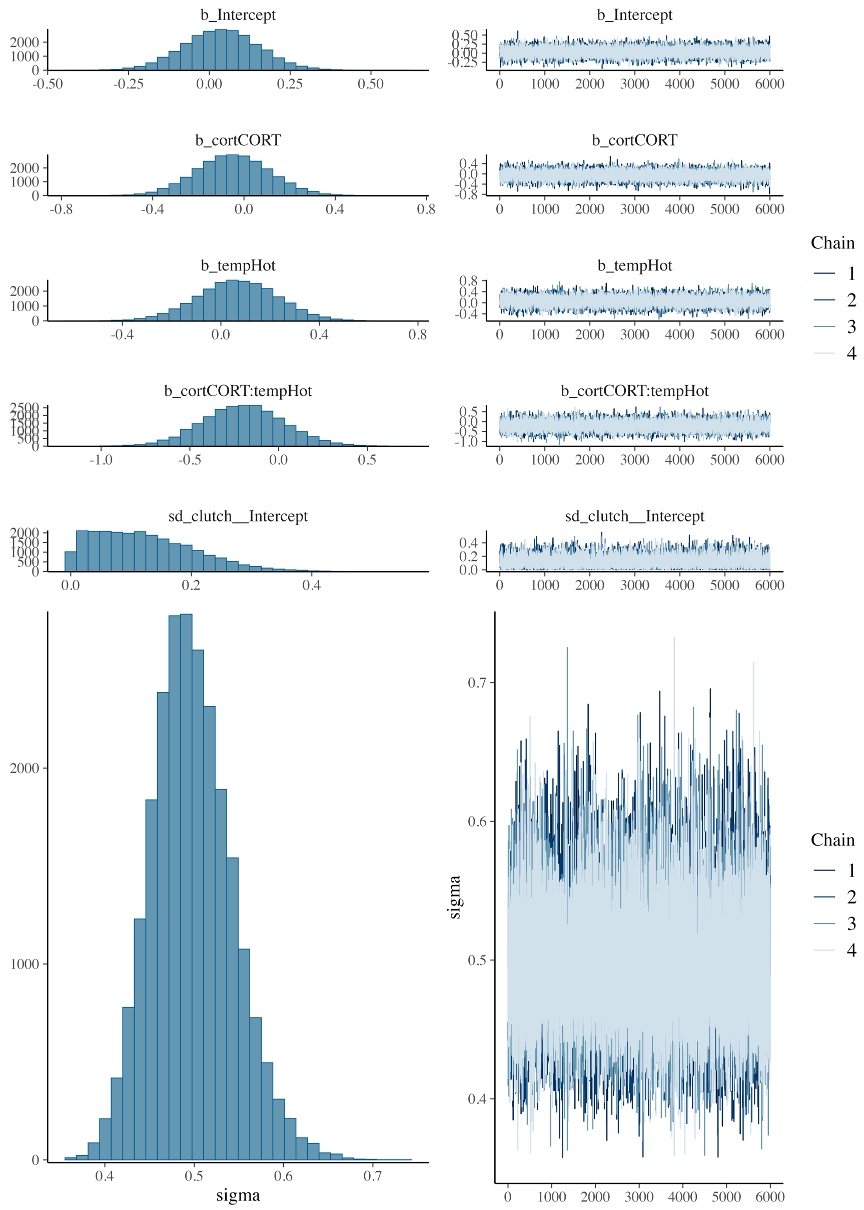

Fig. S4. Posterior predictive checks for the model of ROS. Formula: ROS ~ cort * temp + (1|clutch)

Journal of Experimental Biology • Supplementary information

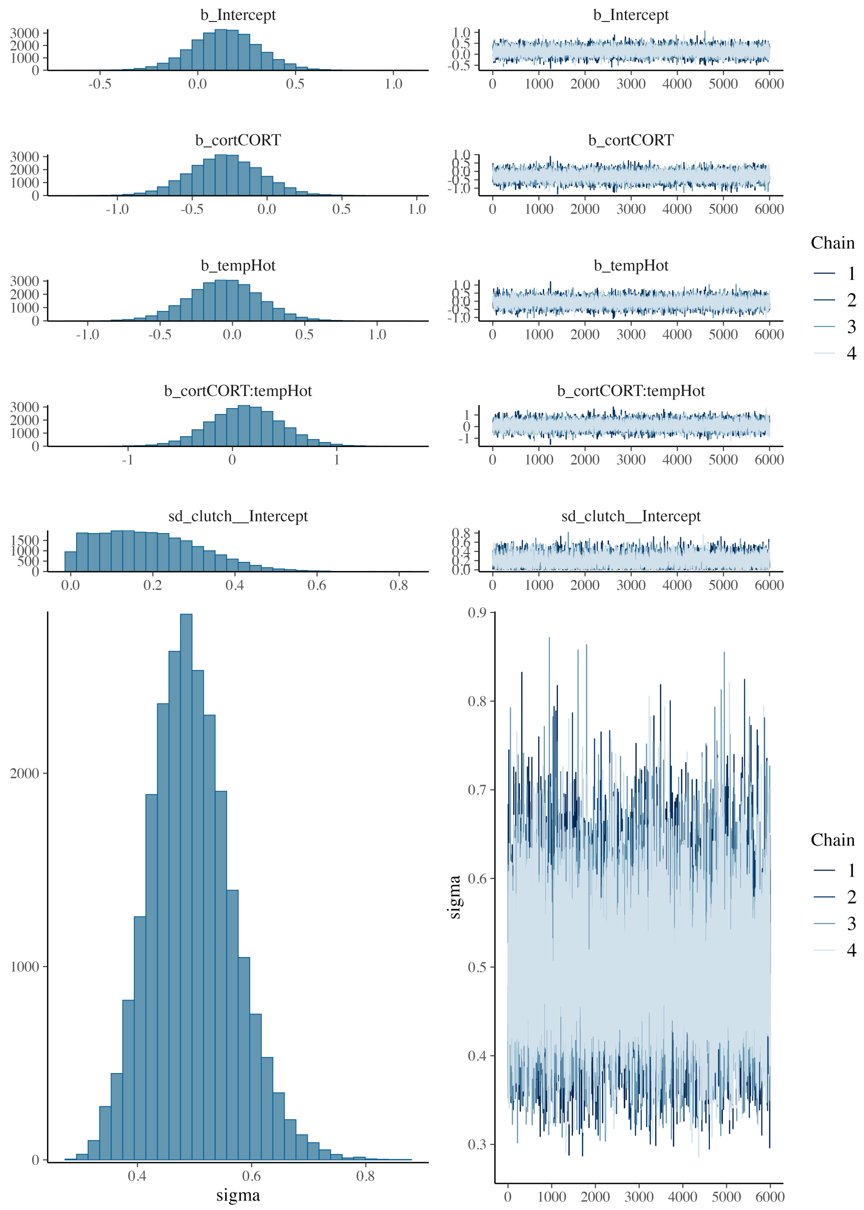

Fig. S5. Posterior predictive checks for the model of DNA Damage. Formula: DNAdamage ~cort * temp + (1|clutch)

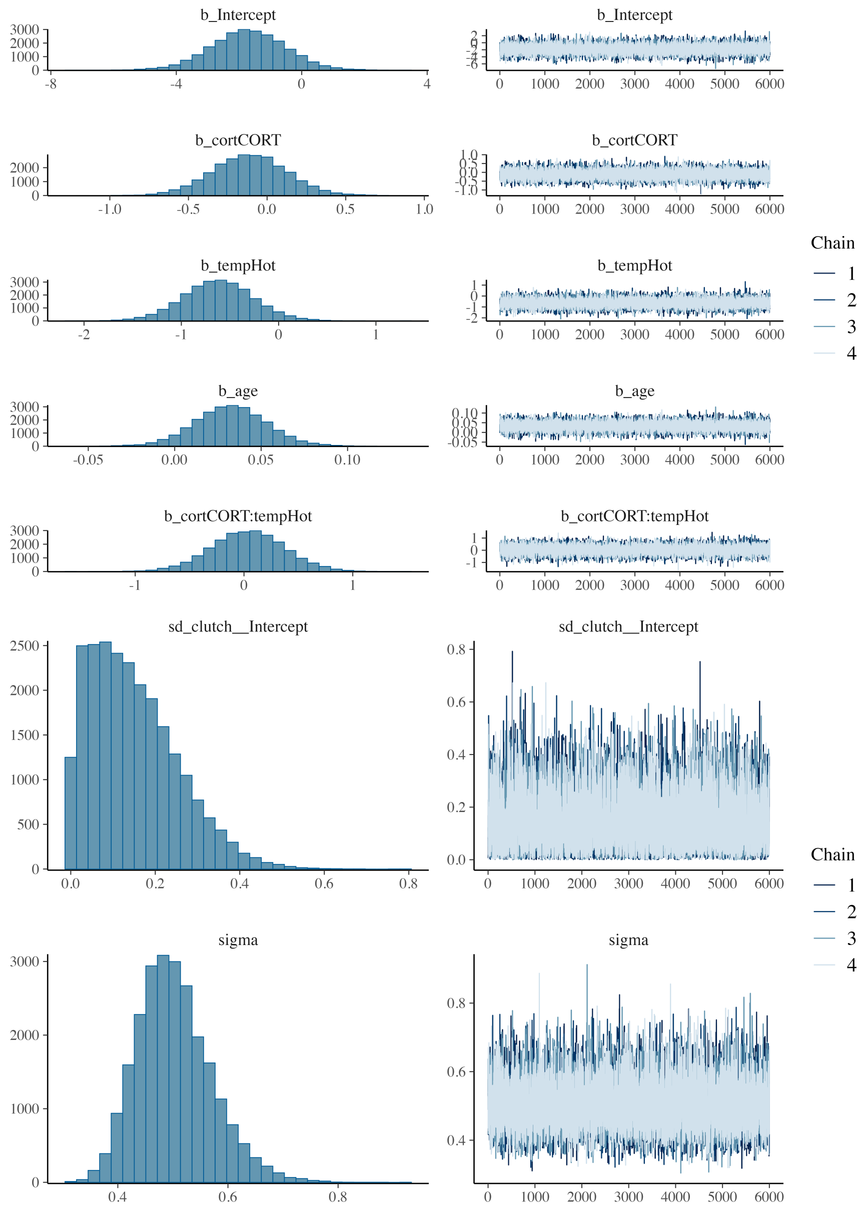

Fig. S6. Posterior predictive checks for the model of lipid peroxidation. Formula: peroxidation ~ cort * temp + (1|clutch)

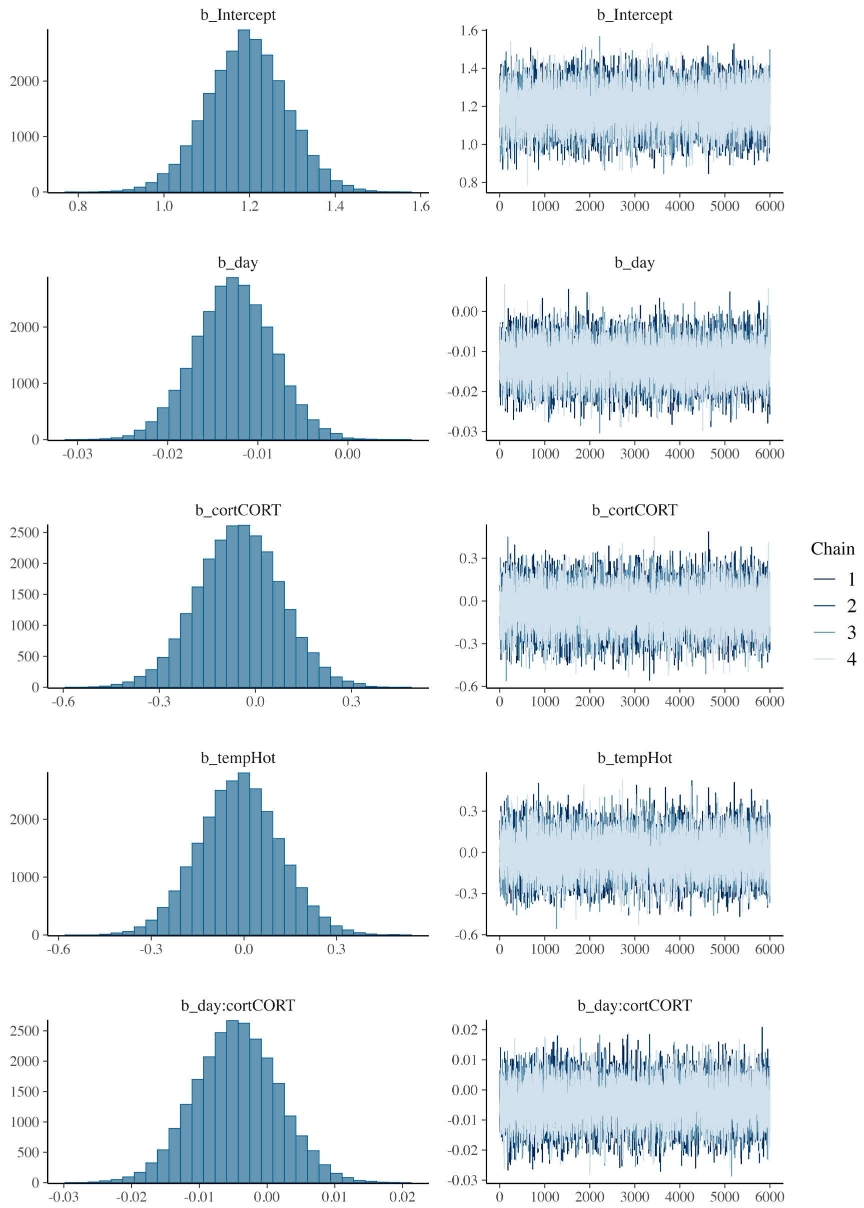
b_Intercept
b_Intercept
b_day
b_day
b_cortCORT
b_cortCORT
b_tempHot
b_tempHot
b_day:cortCORT
b_day:cortCORT
Chain
1
2
3
4
0 1000 2000 3000 4000 5000 6000

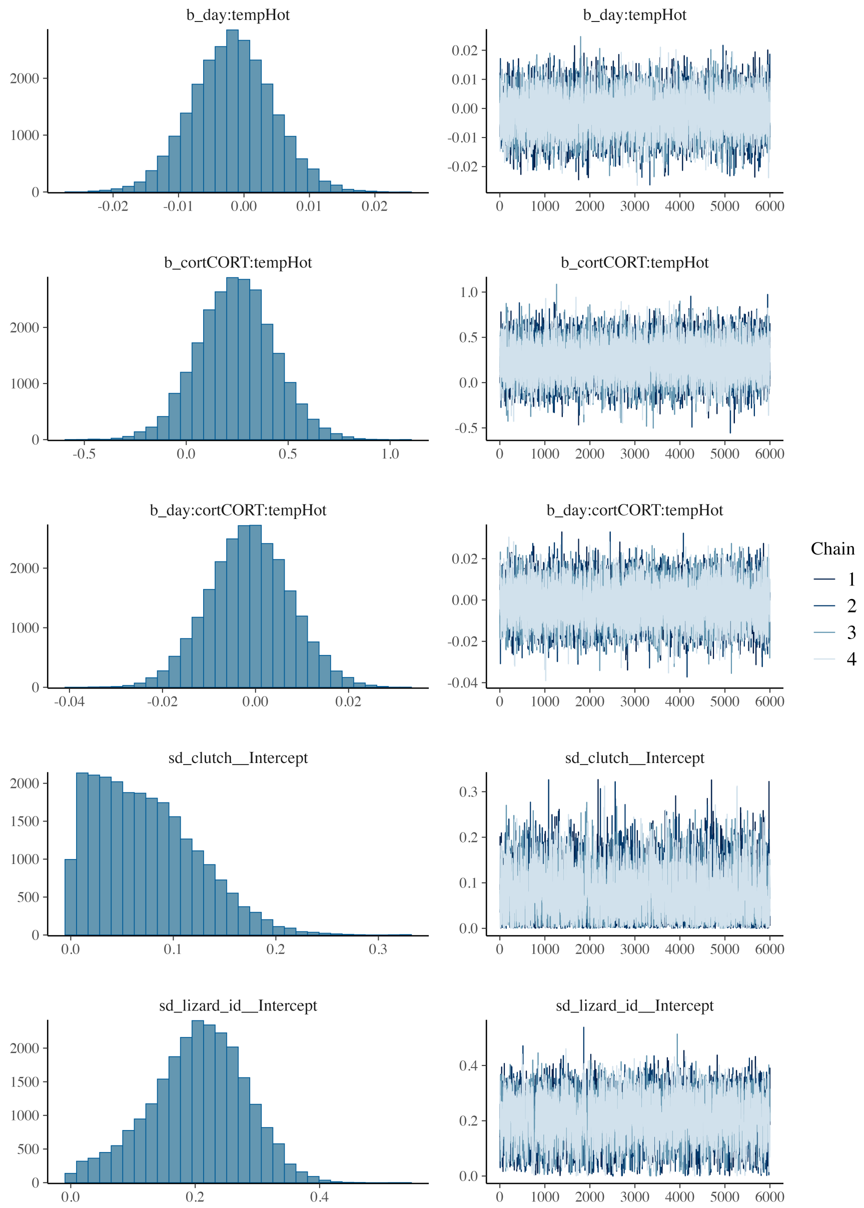

Journal of Experimental Biology • Supplementary information

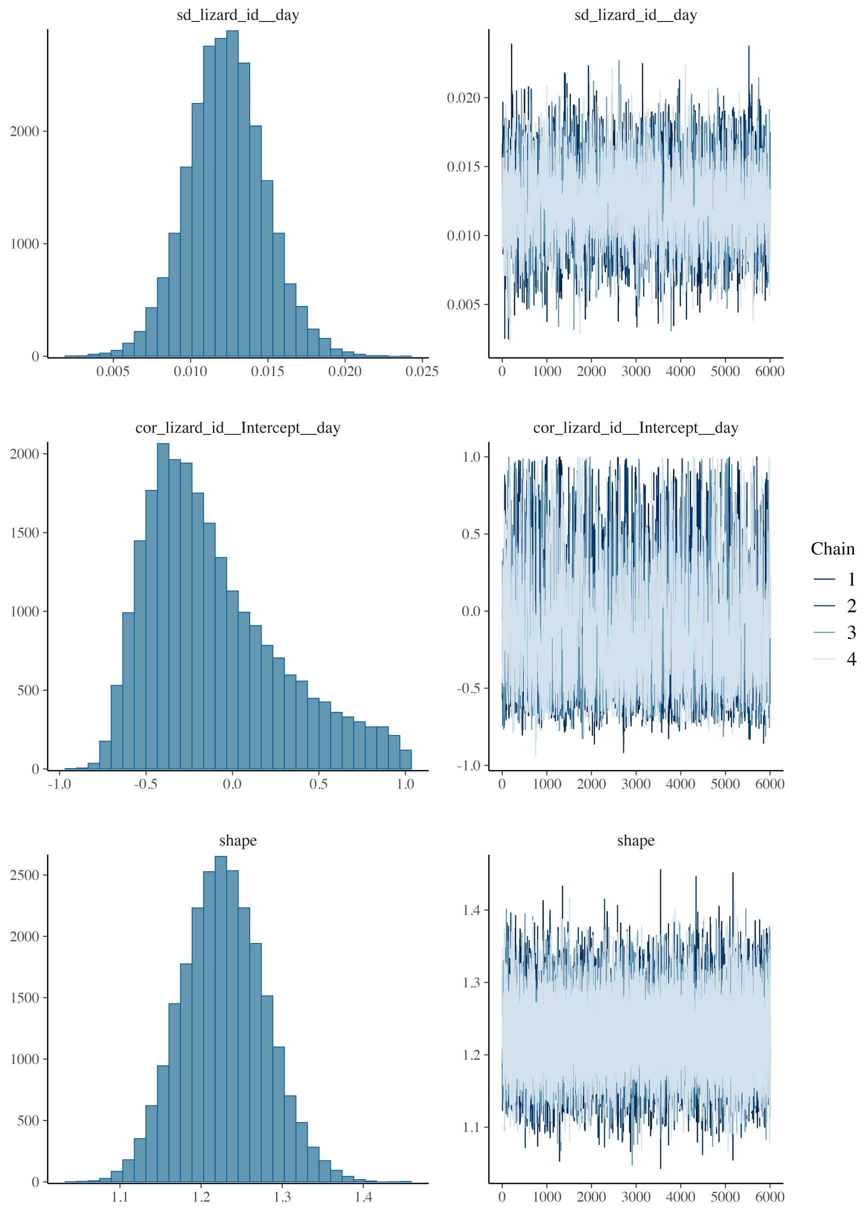

Fig. S7. Posterior predictive checks for the model of spatial learning. Formula: errors ~ day * cort * temp + (1 + day|lizard_id) + (1|clutch)

Journal of Experimental Biology • Supplementary information

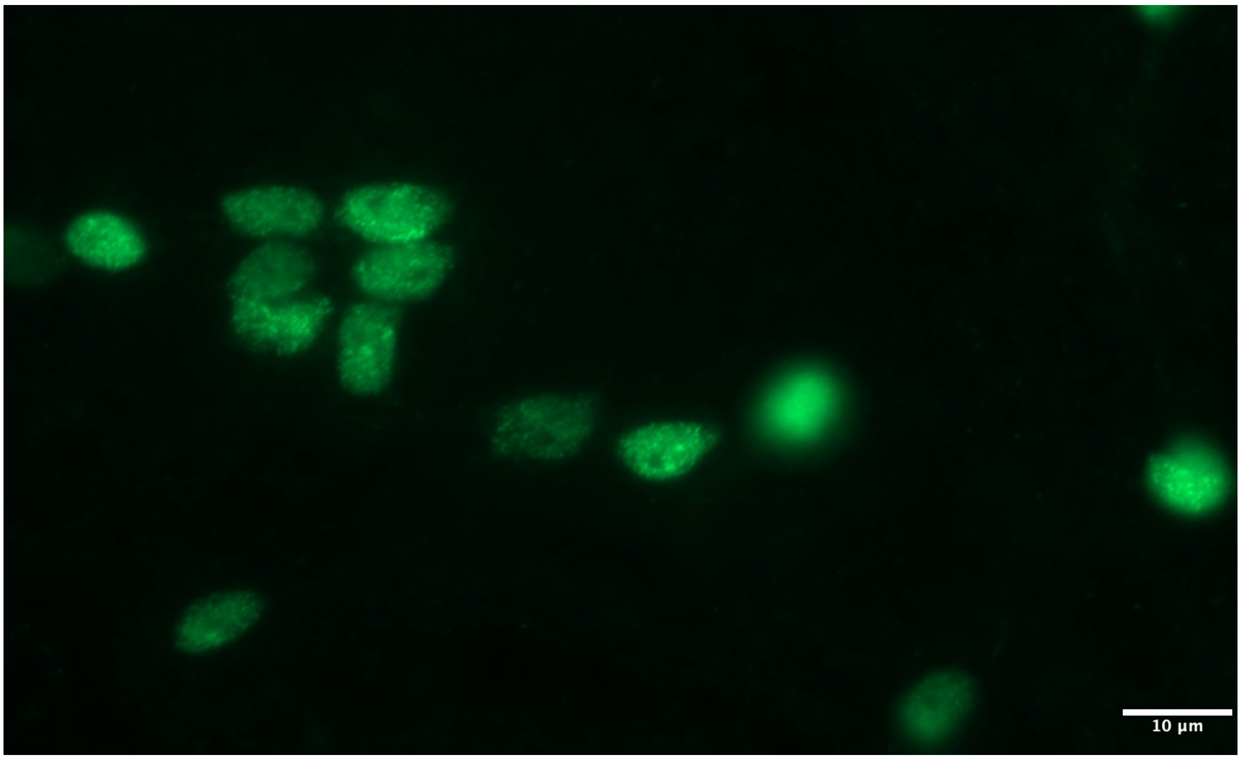

Fig. S8. Fluorescence microscopy image of neuron nuclei stained with NeuN-Alexa 488. Images were taken on a Zeiss AxioObserver Z1, equipped with Zeiss Axiocam 506 monochrome camera. A Zeiss 38HE fluorescent filter set (450-490nm Ex, 500-550nm Em.) was used in conjunction with a 63x 1.4 NA.

Table S1. Estimates of the learning slopes per treatment, the 95% CI, and pMCMC values testing the hypothesis that slope is different from 0. In bold, the values that are significant at pMCMC < 0.05.

Treatment	Estimated slope	95 CI	pMCMC
**CORT-Cold**	**-0.017**	**[-0.026, -0.008]**	**< 0.001**
**CORT-Hot**	**-0.020**	**[-0.029, -0.011]**	**< 0.001**
**Control-Cold**	**-0.013**	**[-0.021, -0.004]**	**< 0.05**
**Control-Hot**	**-0.014**	**[-0.023, -0.006]**	**< 0.05**

Journal of Experimental Biology • Supplementary information

Table S2. Estimate of the contrasts between treatments for all the variables analysed, and pMCMC values testing the hypothesis that contrast is different from 0. In bold, the values that are significant at pMCMC < 0.05.

Variable	Predictor	Contrast	pMCMC contrast
Mit density	Temperature	-0.018	0.917
	CORT	0.134	0.471
	Interaction	0.176	0.437
Metabolic capacity	Temperature	-0.036	0.839
	CORT	0.162	0.364
	Interaction	0.135	0.559
ROS	Temperature	-0.032	0.875
	CORT	0.148	0.450
	Interaction	0.199	0.382
DNA damage	Temperature	0.007	0.970
	CORT	0.217	0.394
	Interaction	-0.128	0.706
Lipid peroxidation	Temperature	-0.601	0.106
	CORT	0.093	0.701
	Interaction	-0.037	0.909
Learning slopes	Temperature	-0.002	0.701
	CORT	0.005	0.416
	Interaction	0.001	0.881

Contrasts were done by:

- *Temperature*: $\beta_{Hot} - \beta_{Cold}$
- *CORT*: $\beta_{CORT} - \beta_{Control}$
- *Interaction*: $(\beta_{Control\text{-}Hot} - \beta_{CORT\text{-}Hot}) - (\beta_{Control\text{-}Cold} - \beta_{CORT\text{-}Cold})$

Table S3. Clutch effect of the final univariate models

Variable	Mean_effect	95% CI
Mit density	0.192	[0.014, 0.398]
Mit potential	0.112	[0.004, 0.293]
ROS	0.123	[0.006, 0.310]
DNA damage	0.191	[0.009, 0.456]
Peroxidation	0.149	[0.006, 0.386]
Learning	0.073	[0.003, 0.183]

Journal of Experimental Biology • Supplementary information

Table S4. Estimated direct, indirect, and total coefficients from the multivariate model

Response	Predictor	Direct effects	Indirect effects	Total effects
Learning	Mitochondrial density	0.068 [-0.270, 0.402]	0.003 [-0.036, 0.049]	0.071 [-0.269, 0.408]
	Metabolic capacity	0.025 [-0.311, 0.358]	-0.012 [-0.124, 0.088]	0.013 [-0.329, 0.357]
	ROS	-	-0.011 [-0.109, 0.080]	-0.011 [-0.109, 0.080]
	DNA damage	0.031 [-0.185, 0.241]	-	0.031 [-0.185, 0.241]
	Lipid peroxidation	-0.119 [-0.324, 0.102]	-	-0.119 [-0.324, 0.102]
DNA damage	Mitochondrial density	-	-0.037 [-0.279, 0.145]	-0.037 [-0.279, 0.145]
	Metabolic capacity	-	0.137 [-0.322, 0.654]	0.137 [-0.322, 0.654]
	ROS	0.125 [-0.292, 0.540]	-	0.125 [-0.292, 0.540]
Lipid peroxidation	Mitochondrial density	-	-0.023 [-0.243, 0.159]	-0.023 [-0.243, 0.159]
	Metabolic capacity	-	0.085 [-0.374, 0.583]	0.085 [-0.374, 0.583]
	ROS	0.076 [-0.335, 0.485]	-	0.076 [-0.335, 0.485]
ROS	Mitochondrial density	-0.282 [-0.932, 0.359]	-	-0.282 [-0.932, 0.359]
	Metabolic capacity	1.090 [0.447, 1.738]	-	1.090 [0.447, 1.738]

Table S5. Preliminary results of the models testing for Mitochondrial Density.

variable	mean	median	sd	mad	q5	q95	rhat	ess_bulk	ess_tail
b_Intercept	0.470	0.460	0.866	0.855	-0.947	1.907	1	19,362.26	16,116.88
b_cortCORT	-0.054	-0.055	0.161	0.160	-0.316	0.211	1	23,841.57	18,938.53
b_tempHot	0.177	0.175	0.255	0.254	-0.240	0.598	1	18,264.83	15,601.67
b_age	-0.008	-0.008	0.015	0.015	-0.033	0.016	1	18,809.92	15,827.44
b_sexm	-0.012	-0.012	0.122	0.122	-0.212	0.187	1	32,791.21	19,114.69
b_cortCORT:tempHot	-0.163	-0.161	0.234	0.231	-0.547	0.219	1	23,053.19	18,337.06

Model formula: mit_density ~ cort * temp + age + sex + (1|clutch). Model convergence was checked through rhat and ess_bulk values. Summary indicates no effect of sex or age, so they were discarded from the final models.

Journal of Experimental Biology • Supplementary information

Table S6. Preliminary results of the models testing for Metabolic capacity.

variable	mean	median	sd	mad	q5	q95	rhat	ess_bulk	ess_tail
b_Intercept	0.302	0.296	0.847	0.839	-1.088	1.697	1	18,682.53	16,852.64
b_cortCORT	-0.095	-0.094	0.166	0.165	-0.369	0.178	1	22,493.38	18,602.43
b_tempHot	0.092	0.091	0.248	0.247	-0.315	0.501	1	18,415.30	16,061.12
b_age	-0.004	-0.004	0.014	0.014	-0.028	0.019	1	18,669.13	16,525.89
b_sexm	0.017	0.016	0.126	0.125	-0.191	0.225	1	31,266.98	17,493.28
b_cortCORT:tempHot	-0.137	-0.137	0.242	0.240	-0.534	0.258	1	21,436.04	16,972.70

Model formula: mit_potential ~ cort * temp + age + sex + (1|clutch). Model convergence was checked through rhat and ess_bulk values. Summary indicates no effect of sex or age, so they were discarded from the final models.

Journal of Experimental Biology • Supplementary information

Table S7. Preliminary results of the models testing for ROS.

variable	mean	median	sd	mad	q5	q95	rhat	ess_bulk	ess_tail
b_Intercept	0.441	0.439	0.845	0.846	-0.938	1.829	1	19,954.88	17,733.51
b_cortCORT	-0.059	-0.058	0.163	0.162	-0.329	0.206	1	21,973.58	19,467.53
b_tempHot	0.160	0.157	0.245	0.245	-0.241	0.565	1	18,615.90	16,581.43
b_age	-0.007	-0.007	0.014	0.014	-0.031	0.016	1	19,777.02	17,300.71
b_sexm	0.021	0.021	0.124	0.123	-0.183	0.224	1	33,495.85	18,274.71
b_cortCORT:tempHot	-0.183	-0.184	0.236	0.235	-0.572	0.203	1	21,404.60	18,058.25

Model formula: ROS ~ cort * temp + age + sex + (1|clutch). Model convergence was checked through rhat and ess_bulk values. Summary indicates no effect of sex or age, so they were discarded from the final models.

Table S8. Preliminary results of the models testing for DNA damage.

variable	mean	median	sd	mad	q5	q95	rhat	ess_bulk	ess_tail
b_Intercept	-0.814	-0.815	1.333	1.286	-2.975	1.384	1	14,193.24	15,015.86
b_cortCORT	-0.286	-0.287	0.249	0.244	-0.695	0.125	1	16,764.42	17,646.72
b_tempHot	-0.278	-0.280	0.402	0.393	-0.936	0.384	1	12,803.98	14,601.59
b_age	0.017	0.017	0.023	0.022	-0.021	0.054	1	13,624.26	14,032.51
b_sexm	0.030	0.031	0.185	0.181	-0.274	0.330	1	20,984.11	17,159.64
b_cortCORT:tempHot	0.108	0.107	0.355	0.350	-0.475	0.690	1	14,479.58	15,703.47

Model formula: DNAdamage ~ cort * temp + age + sex + (1|clutch). Model convergence was checked through rhat and ess_bulk values. Summary indicates no effect of sex or age, so they were discarded from the final models.

Journal of Experimental Biology • Supplementary information

Table S9. Preliminary results of the models testing for lipid peroxidation.

variable	mean	median	sd	mad	q5	q95	rhat	ess_bulk	ess_tail
b_Intercept	-1.960	-1.944	1.280	1.237	-4.098	0.117	1	17,308.48	15,369.02
b_cortCORT	-0.139	-0.139	0.242	0.237	-0.537	0.256	1	17,466.19	16,492.63
b_tempHot	-0.691	-0.691	0.386	0.374	-1.328	-0.056	1	14,335.87	14,037.45
b_age	0.036	0.036	0.022	0.021	0.000	0.073	1	16,551.88	15,440.72
b_sexm	0.119	0.119	0.179	0.175	-0.171	0.414	1	23,967.02	17,240.50
b_cortCORT:tempHot	0.051	0.049	0.337	0.330	-0.508	0.603	1	18,089.57	17,414.62

Model formula: peroxidation ~ cort * temp + age + sex + (1|clutch). Model convergence was checked through rhat and ess_bulk values. Summary indicates no effect of sex, so it was discarded from the final models. However, we saw an effect of age and we included it in our final models.

Table S10. Preliminary results of the models testing for learning.

variable	mean	median	sd	mad	q5	q95	rhat	ess_bulk	ess_tail
b_Intercept	1.350	1.348	0.546	0.537	0.455	2.256	1	14,157.443	16,878.70
b_day	-0.013	-0.013	0.004	0.004	-0.020	-0.005	1	9,398.846	13,567.40
b_cortCORT	-0.054	-0.052	0.134	0.136	-0.275	0.164	1	10,693.409	14,518.00
b_tempHot	0.023	0.022	0.180	0.178	-0.269	0.322	1	10,924.742	15,119.67
b_sexm	-0.005	-0.005	0.080	0.079	-0.136	0.128	1	16,727.891	17,160.45
b_age	-0.003	-0.003	0.009	0.009	-0.018	0.012	1	13,505.073	16,391.78
b_day:cortCORT	-0.004	-0.005	0.006	0.006	-0.015	0.006	1	9,216.274	12,908.06
b_day:tempHot	-0.002	-0.002	0.006	0.006	-0.012	0.008	1	9,945.670	13,744.26
b_cortCORT:tempHot	0.242	0.241	0.194	0.194	-0.076	0.563	1	10,111.700	14,279.42
b_day:cortCORT:tempHot	0.000	0.000	0.009	0.009	-0.015	0.014	1	9,169.257	12,700.42

Model formula: errors ~ day * cort * temp + sex + age+ (1 + day | lizard_id) + (1|clutch). Model convergence was checked through rhat and ess_bulk values. Summary indicates no effect of sex or age, so they were discarded from the final models.

REFERENCES

Farrow, L. F., Andronicos, N. M., McDonald, P. G. and Hamlin, A. S. (2021). Quantitative determination of neuronal size and density using flow cytometry. *Journal of Neuroscience Methods*, **352**, 109081.

Storks, L., Powell, B. J. and Leal, M. (2023). Peeking inside the lizard brain: neuron numbers in Anolis and its implications for cognitive performance and vertebrate brain evolution. *Integrative and Comparative Biology*, **63(1)**, 223-237.

Journal of Experimental Biology • Supplementary information
